# Anatomy Meets Evidence: Left Main Bifurcation Stenting in the Era of Network Meta-analysis

**DOI:** 10.1016/j.jscai.2026.105316

**Published:** 2026-03-31

**Authors:** Eliano P. Navarese, Timothy D. Henry, Dean J. Kereiakes

**Affiliations:** a Department of Life and Health Sciences, Link Campus University, Rome, Italy; b SIRIO MEDICINE Research Network, Department of Cardiology, Nicolaus Copernicus University, Bydgoszcz, Poland; c The Christ Hospital and Lindner Research Center, Cincinnati, Ohio

**Keywords:** angioplasty, bifurcation, left main coronary artery, network meta-analysis, percutaneous coronary intervention

The distal left main coronary artery (LM) remains the epicenter of interventional cardiology—a territory where anatomical complexity, technical precision, and clinical stakes converge with uncommon intensity. Despite decades of refinement in percutaneous coronary intervention (PCI) techniques, the optimal stenting strategy for true LM bifurcation disease remains actively debated, largely because direct head-to-head randomized comparisons across the full spectrum of available techniques are logistically unfeasible. In the current issue of *JSCAI*, Al-Shammari et al^1^ address this evidence gap through a rigorously conducted frequentist network meta-analysis (NMA) incorporating reconstructed time-to-event data—an analytical approach that meaningfully extends the reach of conventional aggregate-level comparisons.

By pooling 15 studies enrolling 3794 patients and applying individual patient-level data reconstruction from published Kaplan-Meier curves, the authors transform digitized survival information into hazard-ratio-based estimates, affording time-varying analyses that simple odds ratios or relative risks cannot provide. This strategy substantially enhances the statistical granularity of the comparisons and partially bridges the gap between observational and randomized data streams within the NMA framework. Notably, the network geometry was strengthened by adequate transitivity, and internal consistency testing through node-splitting analyses showed no major discordance between direct and indirect evidence—features that collectively reinforce confidence in the analytic architecture.

**Figure 1:**
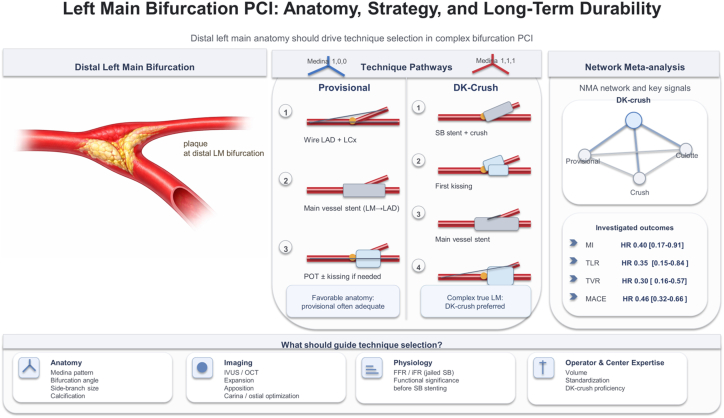
**Left main bifurcation PCI: anatomy, strategy, and long-term durability.** FFR, fractional flow reserve; HR, hazard ratio; iFR, instantaneous wave-free ratio; IVUS, intravascular ultrasound; LAD, left anterior descending coronary artery; LCx, left circumflex artery; LM, left main coronary artery; MACE, major adverse cardiovascular events; MI, myocardial infarction; NMA, network meta-analysis; OCT, optical coherence tomography; PCI, percutaneous coronary intervention; POT, proximal optimization technique; SB, side branch; TLR, target lesion revascularization; TVR, target vessel revascularization.

The central finding is unequivocal: double kissing-crush (DK-crush) emerges as the dominant strategy across the principal efficacy and safety end points, including myocardial infarction, target lesion revascularization, and major adverse cardiovascular events, when compared with provisional stenting, classic crush, and culotte techniques (Figure 1). The magnitude of benefit is particularly striking with respect to stent thrombosis, where the culotte strategy carries a more than 3-fold higher hazard relative to DK-crush. These results converge with—and substantially extend—the landmark DKCRUSH-V trial, which demonstrated target lesion failure rates of 8.3% vs 16.9% at 3 years in favor of DK-crush over provisional stenting, with a pronounced advantage in preventing target vessel myocardial infarction and stent thrombosis,^2^ and with the DKCRUSH-III trial confirming DK-crush superiority over culotte stenting for unprotected distal LM bifurcation lesions.^3^

Yet the editorial lens demands a measured appraisal of what this synthesis cannot resolve. The most prominent methodological constraint is the intrinsic heterogeneity embedded within the pooled population. LM bifurcation lesions span an enormous anatomical spectrum: Medina classification, side branch (SB) ostial involvement, bifurcation angle geometry, and calcification burden all modulate technical challenge and expected outcomes in ways not fully captured by aggregate study-level data. SYNTAX scores—the cornerstone of anatomical risk stratification^4^—were variably reported across included studies, limiting the capacity for anatomically stratified subgroup analyses. This is not a trivial caveat: a technique as technically demanding as DK-crush may demonstrate clear superiority in operator-proficient, high-volume centers, while its benefit-to-risk profile could narrow considerably in settings where procedural volumes are lower, the learning curve longer, and intraprocedural imaging less systematically deployed.

A second limitation pertains to follow-up duration. The NMA captures outcomes across heterogeneous time horizons, and although time-to-event reconstruction partially addresses this, truly long-term data at or beyond 5 years remain sparse within the pooled dataset. Late stent failure, neoatherosclerosis within the bifurcation segment, and the durability of SB patency are biologically plausible concerns at extended follow-up that the present analysis cannot adequately address. Moreover, the nonrandomized nature of the majority of included studies introduces residual confounding that no statistical adjustment can fully neutralize. Operator skill and institutional experience were not randomized, and their differential distribution across techniques likely favors DK-crush groups—where expert operators were more represented—introducing a potential performance bias that warrants explicit acknowledgment.

Of note, a meta-analysis demonstrated that the cardiac mortality benefit of elective coronary revascularization over medical therapy alone is not static but grows incrementally with extended follow-up, reaching statistical significance beyond 5 years.^5^ This time-dependent, compounding survival advantage carries a fundamental implication for distal LM bifurcation PCI: to fully preserve and maximize the long-term mortality benefit that revascularization confers, the initial procedural result must be as durable as possible. Technique optimization—selecting the stenting strategy least prone to target lesion failure and stent thrombosis—and systematic use of intravascular imaging to ensure adequate stent expansion and apposition are, therefore, not ancillary refinements but essential prerequisites for translating the promise of revascularization into sustained clinical benefit. The present NMA, by identifying DK-crush as the technique with the most favorable efficacy and safety profile across the network, provides precisely the type of evidence needed to inform this optimization: the choice of bifurcation strategy at the index procedure is a direct investment in the patient’s long-term prognosis.

The clinical translation is therefore nuanced. DK-crush should not be universally mandated for all LM bifurcation lesions. Provisional stenting, executed with meticulous technique, systematic proximal optimization, and liberal use of intravascular imaging,^6^ retains an important role in anatomically favorable configurations, particularly in Medina 1,0,0 lesions with modest SB involvement and favorable bifurcation angles. Escalation to DK-crush is most strongly justified in true bifurcation lesions with a large and diseased SB (≥2.75 mm), ostial circumflex involvement, and Medina 1,1,1 anatomy, consistent with consensus recommendations from the European Bifurcation Club.^7^

Looking forward, the subfield of distal LM requires prospective registries and ideally randomized platforms specifically designed to compare DK-crush against provisional stenting in contemporary all-comer LM populations, with pre-specified stratification by center volume, operator experience, and imaging guidance modality. Functional assessment of the jailed SB—using fractional flow reserve or instantaneous wave-free ratio—may further refine the decision to stent the SB at all, potentially narrowing the denominator of cases for which complex 2-stent strategies are warranted and aligning procedural choices with physiological reality.^8^ Finally, 2 additional factors may influence LM stenting outcomes: device strategy (including stent design, the growing role of drug-coated balloons [DCB], and the still-uncertain impact of stent strut thickness—particularly in bifurcation PCI, where stent types used across studies were not consistently identified) and the type and duration of antiplatelet therapy.

In summary, the work by Al-Shammari et al represents a methodologically sophisticated and clinically meaningful contribution to the LM bifurcation literature. By harnessing the power of time-to-event data reconstruction within a comprehensive NMA, it delivers the most granular evidence synthesis to date, favoring DK-crush as the preferred percutaneous strategy for anatomically complex distal LM bifurcation disease. Its conclusions, however, should be interpreted not as a mandate for uniform adoption, but as a compelling argument for structured operator training, rigorous case selection informed by anatomical and physiological imaging, and sustained investment in the randomized evidence base that this highest-stakes territory of interventional cardiology deserves.
